# Literary Notes

**Published:** 1899-04

**Authors:** 


### LITERARY NOTES.

The Grosvenor library, as has been heretofore announced in the Journal, has successfully launched its medical department, and the distribution of the printed catalogues of this branch to the practising physicians of Buffalo has been completed. Anyone failing to receive a copy can secure one by sending such name and address to the library.

Physicians wishing extracts made from the medical works can have this done by the young woman in charge of the balcony floor of the library. As her time for such work must be limited by her other duties, it may not be possible to supply copies of all that is wanted, but so far as this can be done it will be done willingly and gratuitously.

Another arrangement has been made by the trustees of the Grosvenor Library that it is hoped will be appreciated by the physicians of this city. In this department a book donated to the library may be taken out at any time by the donor, or on his written order, for a period of one or two weeks at a time. It is hoped that under this arrangement physicians will be enabled to place many books that they use for occasional reference only, in the Grosvenor Library where others may have the benefit of their use.

---

The International Medical Annual for 1899 is announced. It is a work of reference for medical practitioners, alphabetically arranged, and combines the features of an annual retrospect with those of a medical encyclopedia. It is copiously illustrated with elegant plates, in colors and black and white. Each volume contains entirely new matter. Price, $3 net, post or express paid. E. B. Treat & Co. are the publishers, 241 to 243 West 23d street, New York.

